# Oxidant sensor in the cGMP-binding pocket of PKGIα regulates nitroxyl-mediated kinase activity

**DOI:** 10.1038/s41598-017-09275-1

**Published:** 2017-08-30

**Authors:** Sonia Donzelli, Mara Goetz, Kjestine Schmidt, Markus Wolters, Konstantina Stathopoulou, Simon Diering, Oleksandra Prysyazhna, Volkan Polat, Jenna Scotcher, Christian Dees, Hariharan Subramanian, Elke Butt, Alisa Kamynina, Sophie Schobesberger, S. Bruce King, Viacheslav O. Nikolaev, Cor de Wit, Lars I. Leichert, Robert Feil, Philip Eaton, Friederike Cuello

**Affiliations:** 1 Department of Experimental Pharmacology and Toxicology, Cardiovascular Research Center, University Medical Center Hamburg-Eppendorf, Martinistrasse 52, 20246 Hamburg, Germany; 2 DZHK (German Center for Cardiovascular Research), partner site Hamburg/Kiel/Lübeck, University Medical Center Hamburg-Eppendorf, Martinistrasse 52, 20246 Hamburg, Germany; 3 Institute of Physiology, University of Lübeck, Ratzeburger Allee 160, 23562 Lübeck, Germany; 4 Interfaculty Institute of Biochemistry, University of Tübingen, Hoppe-Seyler-Str. 4, 72076 Tübingen, Germany; 5 King´s College London, Cardiovascular Division, British Heart Foundation Centre of Excellence, the Rayne Institute, St Thomas’ Hospital, London, SE17EH United Kingdom; 6 Institute of Experimental Biomedicine II, University Medical Center Würzburg, Grombühlstraße 12, 97080 Würzburg, Germany; 7 Institute of Experimental Cardiovascular Research, University Medical Center Hamburg-Eppendorf, Martinistrasse 52, 20246 Hamburg, Germany; 8 Department of Chemistry, Wake Forest University, Winston-Salem, North Carolina 27109 USA; 9 Institute of Biochemistry and Pathobiochemistry - Microbial Biochemistry, Ruhr University Bochum, Universitätsstrasse 150, 44780 Bochum, Germany; 10 Present Address: Laboratory of Molecular and Chemical Biology of Neurodegeneration, Brain Mind Institute, Ecole Polytechnique Fédérale de Lausanne (EPFL), 1015 Lausanne, Switzerland

## Abstract

Despite the mechanisms for endogenous nitroxyl (HNO) production and action being incompletely understood, pharmacological donors show broad therapeutic promise and are in clinical trials. Mass spectrometry and site-directed mutagenesis showed that chemically distinct HNO donors 1-nitrosocyclohexyl acetate or Angeli’s salt induced disulfides within cGMP-dependent protein kinase I-alpha (PKGIα), an interdisulfide between Cys42 of the two identical subunits of the kinase and a previously unobserved intradisulfide between Cys117 and Cys195 in the high affinity cGMP-binding site. Kinase activity was monitored in cells transfected with wildtype (WT), Cys42Ser or Cys117/195Ser PKGIα that cannot form the inter- or intradisulfide, respectively. HNO enhanced WT kinase activity, an effect significantly attenuated in inter- or intradisulfide-deficient PKGIα. To investigate whether the intradisulfide modulates cGMP binding, real-time imaging was performed in vascular smooth muscle cells expressing a FRET-biosensor comprising the cGMP-binding sites of PKGIα. HNO induced FRET changes similar to those elicited by an increase of cGMP, suggesting that intradisulfide formation is associated with activation of PKGIα. Intradisulfide formation in PKGIα correlated with enhanced HNO-mediated vasorelaxation in mesenteric arteries *in vitro* and arteriolar dilation *in vivo* in mice. HNO induces intradisulfide formation in PKGIα, inducing the same effect as cGMP binding, namely kinase activation and thus vasorelaxation.

## Introduction

Nitroxyl (HNO) is the one-electron-reduced and protonated sibling of nitric oxide. The extent to which HNO is produced endogenously and the mechanisms of its generation are still under intense investigation^1, 2^. Despite this, pharmacological donors that release HNO have emerged as promising therapeutic reagents, with clinical trials ongoing^3–5^. The beneficial actions attributed to experimental therapeutic HNO release are multifaceted, such as vasorelaxation of human conduit and resistance arteries and lowering of blood pressure^6, 7^, relief from chronic neuropathic pain^8^, anti-tumour effects^9^ and positive cardiac inotropy and lusitropy^10, 11^. Unlike nitric oxide or some routine clinically-utilised vasodilatory nitrates, HNO is not susceptible to vascular tolerance^6^, is resistant to scavenging by reactive oxygen species^12^, and maintains its effects also under pathophysiological conditions^13–15^. Several mechanisms for HNO-mediated vasorelaxation have been suggested, including activation of soluble guanylyl cyclase (sGC) to promote classical cGMP-dependent signaling^16^, opening of voltage and calcium-dependent potassium channels^7^ and release of calcitonin gene-related peptide^17^. However, the sGC inhibitor 1H-(1,2,4)oxadiazolo-(4,3-a)quinoxalin-1-one (ODQ) only partially attenuated HNO-mediated vasorelaxation in aortic rings^14^, and *in vivo* administration of HNO reduced systemic blood pressure without increasing plasma cGMP levels^18^. Therefore, the exact mechanisms of how HNO induces vasorelaxation and what targets in the vasculature mediate this are incompletely understood and require further investigations.

cGMP-dependent protein kinase (PKG) is a homodimeric serine/threonine kinase involved in numerous processes, including regulation of smooth muscle tone and vasorelaxation^19^. The isoform I-alpha (PKGIα) is expressed in the vascular system^20^, it consists of an N-terminal leucine zipper, an autoinhibitory domain, a low and a high affinity cGMP-binding domain and a C-terminal catalytic domain. Classical PKGIα activation occurs upon cGMP binding to regulatory sites, with the release of autoinhibition as a consequence^21, 22^. In addition to this, the kinase can be oxidized at cysteine 42 (Cys42), resulting in an intermolecular disulfide bond, which contributes to cGMP-independent targeting and activation of the kinase^23, 24^. However, oxidation of Cys42 may not induce substantive catalytic activation compared to cGMP-dependent activation of the kinase^25^. Given that Cys42 is localised in the N-terminal leucine zipper domain that is important for substrate targeting^26^, the redox state of this thiol may be more important for modulating such interactions, as suggested previously^27–29^. Consistent with this regulatory role, Cys42Ser PKGIα knock-in (KI) mice, which cannot form the interprotein disulfide, are hypertensive^30^, illustrating a physiological role for oxidants in blood pressure regulation.

In addition to Cys42, PKGIα possesses other cysteines that may play a role in regulating kinase activity. Among them, Cys117 and Cys195, localised in the high affinity cGMP-binding site have been shown to form an intradisulfide in the crystal structure of PKGIα^24, 31^. This intradisulfide in the second messenger-binding site represents a more rational explanation for modulating kinase activity in response to oxidants than the interdisulfide in the leucine zipper domain. However, whether oxidation of these cysteines plays a role in regulation of PKGIα activity is, to date, unknown.

## Materials and Methods

Anti-PKG antibody was from Enzo Life Sciences (♯ADI-KAP-PK005-D; Lörrach, Germany). NCA was synthesised by S. Bruce King^32, 33^ or by Axon Medchem (Groningen, The Netherlands). AS (Na_2_N_2_O_3_) was obtained from Cayman Europe (Tallinn, Estonia). Acetylcholine (ACh) was from Sigma-Aldrich (Taufkirchen, Germany) and NOxICAT kit from AB Sciex (Concord, ON, Canada). Ni-NTA agarose beads were from Qiagen (Hilden, Germany). PKGIα from bovine lung (obtained from Südfleisch GmbH, Würzburg, Germany) was purified as previously described^34^ in accordance to the European TierSchlV No.1099/2009. γ^32^P-ATP was purchased from GE Healthcare (Freiburg, Germany) and (^3^H)cGMP from American Radiolabeled Chemicals (USA). Glass microfiber filters were purchased from Whatman (Maidstone, UK). Complete protease inhibitor cocktail was from Roche (Berlin, Germany). Human embryonic kidney cells (HEK-293) were purchased from ATCC (293-(HEK-293)(ATCC^©^CRL-1573^TM^); Wesel, Germany).

All methods were carried out in accordance with the relevant guidelines and regulations. Experiments were approved by the relevant institutional or licensing committees as indicated in the relevant section.

### Transfection

HEK-293 cells were used for transfection experiments. Cells were routinely screened for mycoplast contaminations. Cells were transfected with empty pcDNA3 as a control or pcDNA3 containing the cDNA for human WT, Cys42Ser, Cys117Ser, Cys195Ser, Cys117/195Ser or Cys42/117/195Ser PKGIα (2 µg DNA/well) using Turbofect (Turbofect, Thermo Scientific, Venlo, Limburg, The Netherlands). After 24 hours, cells were treated with NCA (100 µmol/L, 30 min), AS (500 µmol/L, 15 min) or vehicle (1% DMSO or 100 µmol/L decomposed NCA for NCA; 10 mmol/L NaOH, 50 µmol/L nitrite (NO_2_^−^), 500 µmol/L decomposed AS for AS). Western immunoblot analysis was performed to assess oxidative PKGIα modifications or *in vitro* kinase assays to assess PKGIα activity.

### Western immunoblot analysis

Snap frozen isolated mesenteric vessels (after 100 µmol/L NCA), cremaster muscles (after 50 µmol/L NCA or AS) or HEK-293 cell homogenates (after 100 µmol/L NCA or 500 µmol/L AS) in non-reducing (containing in mmol/L: Tris-HCl 187.5 pH 6.8, SDS 6% (w/v), glycerol 30% (v/v), bromophenol blue 0.03% (w/v), maleimide 100) or reducing (9% (w/v) 2-mercaptoethanol) Laemmli sample buffer were subjected to western immunoblot analysis as described previously^35^.

### NOxICAT LC-MS/MS analysis

Modified cysteines in PKGIα by HNO were identified by a thiol-trapping technique using isotope-coded affinity-tag chemistry (NOxICAT) as described previously^36^. Briefly, non-tagged recombinant human PKGIα^29^ was pre-treated with DTT (10 mmol/L, 30 min) in an anaerobic chamber, the DTT was removed by buffer exchange via passing the sample through a PD MiniTrapTM G-25 column (28-9180-07) from GE Healthcare. After buffer exchange, samples were exposed to NCA (25 µmol/L, 15 min), AS (25 µmol/L, 15 min) or vehicle for 15 min at RT (Supplementary Figure 1A,B). The reaction was terminated by addition of ice-cold acetone and proteins precipitated overnight at −20 °C. The precipitate was resuspended in a mixture of 80 µL denaturing alkylation buffer (DAB; containing in mmol/L: urea 6000, Tris-HCl 200 pH 8, EDTA 10, SDS 0.5% (w/v)) containing 20 µL acetonitrile and 1 vial ICAT reagent “light” from the cleavable ICAT methods development kit (AB Sciex). Reduced cysteines were labeled under denaturing conditions at 37 °C in a thermomixer at 1300 rpm for 2 hours. After a second acetone precipitation, oxidised cysteines were reduced for 30 min in 80 µL DAB containing 2 mmol/L TCEP. Subsequently, 1 vial cleavable ICAT reagent “heavy”, resuspended in 20 µL acetonitrile, was added and previously oxidised cysteines were labeled at 37 °C at 1300 rpm for 2 hours. The reaction was stopped by acetone precipitation. The pellet was resuspended in 80 µL denaturing buffer from the cleavable ICAT reagent kit. 20 µL acetonitrile was added to the protein digest with 100 µL aqueous trypsin resuspension from the ICAT reagent kit. ICAT labeled peptides were purified by cation exchanger chromatography and affinity chromatography. Light- and heavy-ICAT-labeled peptides were analysed and quantified by reverse phase nano-liquid chromatography and detected by MS/MS with Fourier transform mass spectrometry in an LTQ Orbitrap instrument (Thermo Fisher Scientific, Waltham, MA). Peptides were identified by SwissProt database and quantified by MaxQuant.

### FRET-experiments in primary vascular smooth muscle cells

The isolation of primary mouse vascular smooth muscle cells (VSMCs) from cGi500 transgenic mice was approved by the Regierungspräsidium Tübingen in compliance with the humane care and use of laboratory animals. For in-cell FRET measurements, primary VSMCs from R26-CAG-cGi500(L1) mice^37^ were used. These cells express the FRET-based biosensor cGi500 (cGMP indicator with an EC_50_ of 500 nmol/L)^38^. VSMCs were continuously superfused with intracellular-like medium (ICM; containing in mmol/L: HEPES 10 pH 7.3, KCl 125, NaCl 19, EGTA 1, CaCl_2_ 0.3) at room temperature. Cells were permeabilised by superfusion with β-escin (100 µmol/L in ICM for 80 sec) and subsequently exposed to ICM supplemented with increasing concentrations of cGMP (0.1, 1, 10 µmol/L; 2 min each). The individual fluorescence of CFP and YFP was recorded simultaneously using a DualView DV^2^ beamsplitter (Photometrics) and saved as individual TIFF images. After each cGMP application, baseline recovery of the fluorescence signals was achieved before the next drug application. After the initial series of cGMP applications, VSMCs were exposed to 0.02% DMSO (in ICM) or 100 µmol/L NCA (in ICM containing 0.02% DMSO) for 30 min followed by superfusion with ICM supplemented with increasing concentrations of cGMP (0.1, 1, 10 µmol/L). Acquired images were analysed using Fiji^39^ and Microsoft Excel to correct for background fluorescence and calculate the CFP/YFP ratio (R) traces, which reflect FRET changes. FRET responses were measured as amplitudes over baseline. The individual FRET changes (ΔR) were then normalised to the FRET change induced by the first application of 10 µmol/L cGMP (ΔR0) and are displayed as ΔR/ΔR0. Further details on measurement and evaluation of FRET signals in VSMCs are described elsewhere^37, 40^.

### *In vitro* cGMP-binding assay

cGMP-binding affinity to purified bovine PKGIα was assessed by measuring (^3^H)cGMP binding in the presence of increasing concentrations of unlabeled cGMP. The experiment was performed under ambient air. PKGIα (600 fmol/reaction) was diluted in cGMP-binding buffer (containing in mmol/L: HEPES 6.7 pH 7.4, Mg(CH_3_COO)_2_ 5, NaH_2_PO_4_ 2H_2_O, 3, KCl 130, IBMX 0.1, EGTA 0.1, EDTA 0.1). PKGIα was either reduced with DTT (100 mmol/L, 10 min) or oxidised with NCA (100 μmol/L, 30 min) followed by incubation with (^3^H)cGMP in the presence of increasing concentrations of unlabeled cGMP (0, 10, 30, 100, 300, 1000 nmol/L) for 1 h. Reactions were terminated by adding ice-cold saturated ammonium sulfate buffer (pH 8.3) and vacuum-filtered through glass microfiber filters (⦸ 25 mm). The filter papers were washed three times with 2 mL ice-cold ammonium sulfate buffer and air-dried. Papers were put into scintillation vials and suspended in 2 mL 2% SDS (w/v), shaken vigorously and incubated for 1 h at room temperature. Afterwards, 10 ml aqueous scintillation liquid (Rotiszint® Eco plus from Carl Roth) was added and incubated for 10 min prior to scintillation counting.

### *In vitro* PKG activity assay

Protein kinase activity was investigated using two different assay systems. On the one hand, activity of purified bovine PKGIα was assessed by Glasstide (Calbiochem) phosphorylation in the presence of radiolabeled γ^32^P-ATP (GE Healthcare). The experiment was performed under ambient air. PKGIα (600 fmol/reaction) was initially reduced with DTT (100 mmol/L, 10 min) or oxidised with NCA (100 μmol/L, 15 min) and *in vitro* kinase assays performed in assay buffer (containing in mmol/L: Tris 30 pH 7.4, ATP 0.1, MgCl_2_ 15, Glasstide 0.1), in the absence or presence of 300 nmol/L cGMP. Reactions were terminated with phosphoric acid (25 mmol/L) before spotting onto P81 phosphocellulose squares (Whatman). After air-drying, the filter papers were washed with phosphoric acid (75 mmol/L; 4 × 2 min), ethanol (1 × 15 sec), air-dried and transferred into scintillation vials. Vials were subjected to Cerenkov counting. Six independent experiments were carried out. PKGIα activity was expressed as pmol phosphate incorporated into PKGIα substrate per minute. On the other hand, activity of human WT, Cys42Ser, Cys117/195Ser, or Cys42/117/195Ser PKGIα was assessed by phosphorylation of recombinantly expressed His_6_-tagged cardiac myosin-binding protein C (amino acid residues 153-450)^41^, which was previously described as a PKGIα substrate^42^, in the presence of radiolabeled γ^32^P-ATP (GE Healthcare). HEK-293 cells were transfected as described before and exposed to AS (500 µmol/L, 15 min), NCA (100 µmol/L, 30 min) or vehicle (NaOH for AS; DMSO for NCA). Cell homogenates were prepared in lysis buffer (containing in mmol/L: Tris 20 pH 7.4, NaCl 150, EDTA 1, EGTA 1, NaF 2, Complete protease inhibitor cocktail) and were diluted 1:1 in assay buffer (containing in mmol/L: Tris 30 pH 7.4, MgCl_2_ 15) with ATP (30 µmol/L spiked with γ^32^P-ATP (GE Healthcare)). The *in vitro* kinase reaction was started by addition of His_6_-tagged C1-M-C2 (500 pmol/reaction) prebound to Ni-NTA agarose beads equilibrated in assay buffer. The reaction was carried out for 30 min at 30 °C and 1300 rpm. Samples were centrifuged at 4 °C for 1 min at 1000 xg, supernatant discarded and the agarose beads resuspended in 75 µL 3x reducing Laemmli sample buffer, heated for 5 min at 75 °C and proteins resolved by 10% SDS-PAGE. Gels were stained with colloidal coomassie to assure equal substrate content between samples, destained in 20% (v/v) methanol, incubated briefly in 20% (v/v) glycerol in water and vacuum-dried. Experiments were analysed by autoradiography and densitometry was performed with GelQuant.NET software provided by biochemlabsolutions.com.

### Myography

All animal protocols were approved by the local King’s College London UK Ethical Review Process Committee and by the UK Government Home Office (Animals Scientific Procedures Group) and the study was conducted in accordance with the Home Office Guidance on the Operation of the Animals. Third-order mesenteric arteries from 12-week-old male C57BL/6 mice were mounted for isometric tension recordings in a pressure myograph (Danish Myo Technology), stretched to the optimal pre-tension conditions (using DMT Normalisation Module), bathed in Krebs solution maintained at 37 °C and gassed with 95% CO_2_: 5% O_2_ (containing in mmol/L: NaCl 119, KCl 4.7, KH_2_PO_4_ 1.2, NaHCO_3_ 25, MgSO_4_*7H_2_O 1.2, glucose 11.1, CaCl_2_*2H_2_O 1.6). Vasorelaxation of mesenteric arteries was assessed after U46619-pre-constriction (100 nmol/L) in response to HNO donors (NCA: 0–300 μmol/L or AS: 0–100 µmol/L). The concentration-response curves to NCA were constructed in a cumulative fashion. Tension experiments were carried out using one or two vessels per intervention derived from at least 4 different WT animals.

### Intravital microscopy

12-week-old male C57BL/6NCrl mice (Charles River) were used for intravital microscopy of the microcirculation in the cremaster muscle *in vivo* as described previously^43^. Experiments were in accordance with the German animal protection law and approved by the Ministerium für Energiewende, Landwirtschaft, Umwelt und ländliche Räume of Schleswig-Holstein. Mice were anaesthetised with fentanyl (0.05 mg/kg), midazolame (5 mg/kg), and dexmedetomidine (0.5 mg/kg) by intraperitoneal injection. After insertion of a catheter into the right jugular vein, anesthetic drugs were continuously infused. A tube was inserted via tracheotomy and the animals were ventilated with a stroke volume of 0.225 mL at 160 strokes per minute using a respirator (MiniVent, Harvard Apparatus). The right cremaster muscle was exposed and spread over a coverslip to allow intravital microscopy. It was continuously superfused with a 35 °C warmed saline solution (containing in mmol/L: NaCl 118.4, KCl 3.8, CaCl_2_ 2.5, MgSO_4_ 1.2, NaHCO_3_ 20, KH_2_PO_4_ 1.2) with a pH of 7.4 achieved by gassing with 5% CO_2_ in N_2_. In each mouse 7 to 18 arterioles were studied using an optical microscope (Eclipse E600, Nikon) and a 20-fold objective. The microscope was equipped with a digital camera (Zeiss Axiocam 105) connected to a PC to allow image storage (Zen2 lite, Zeiss) and later offline analysis. Arteriolar inner diameters were measured by a code written in the laboratory using the commercially available software (LabVIEW, National Instruments). Arteriolar diameters were assessed before and during application of NCA (10 or 50 µmol/L; group 1) or AS (3 to 50 µmol/L; group 2). The respective solvent (0.1% DMSO for NCA or 0.1 mmol/L NaOH for AS) was also evaluated. To assess vascular reactivity, the effect of acetylcholine (ACh, 10 µmol/L) was also assessed. All substances were added to the superfusion solution using a roller pump and the final concentration on the preparation is indicated. After application of each substance, vessels were allowed to recover for 3 to 5 min and to return to their resting diameter. At the end of the experiment, mice were sacrificed by intravenous injection of pentobarbital (24 mg) and both cremaster muscles of each animal were harvested for western blot analysis. While the right cremaster muscle was exposed to the respective substances during the experiment, the left cremaster muscle was prepared immediately after pentobarbital injection and served as untreated control for western immunoblot analysis.

### Statistical analysis

Statistical comparisons were performed by one-way ANOVA (*in vitro* kinase assays with HEK-293 lysates) or two-way ANOVA *(in vitro* kinase assays with bovine PKGIα; FRET analysis) followed by Bonferroni’s multiple comparisons test; Student’s *t*-test comparing samples with the same cGMP concentration (cGMP-binding assays). Arteriolar diameter changes are normalised to the respective maximal possible response:

$$ \% \,\,{\rm{of}}\,{\rm{maximal}}\,{\rm{response}}=({{\rm{D}}}_{{\rm{Subst}}}\mbox{--}{{\rm{D}}}_{{\rm{Con}}})/({{\rm{D}}}_{{\rm{Max}}}\mbox{--}{{\rm{D}}}_{{\rm{Con}}})\times 100$$

where D_Subst_ is the diameter in the presence of the substance, D_Con_ the control diameter before application and D_Max_ the respective maximal diameter observed for each vessel during the experiment. Data within groups were compared using paired *t*-test and corrected according to Bonferroni for multiple comparisons. Quantitative data are given as mean ± S.E.M and a value of *P* < 0.05 was considered significant.

### Data availability

The authors declare that all data supporting the findings of this study are available within the paper and its supplementary file.

## Results

**Figure 1:**
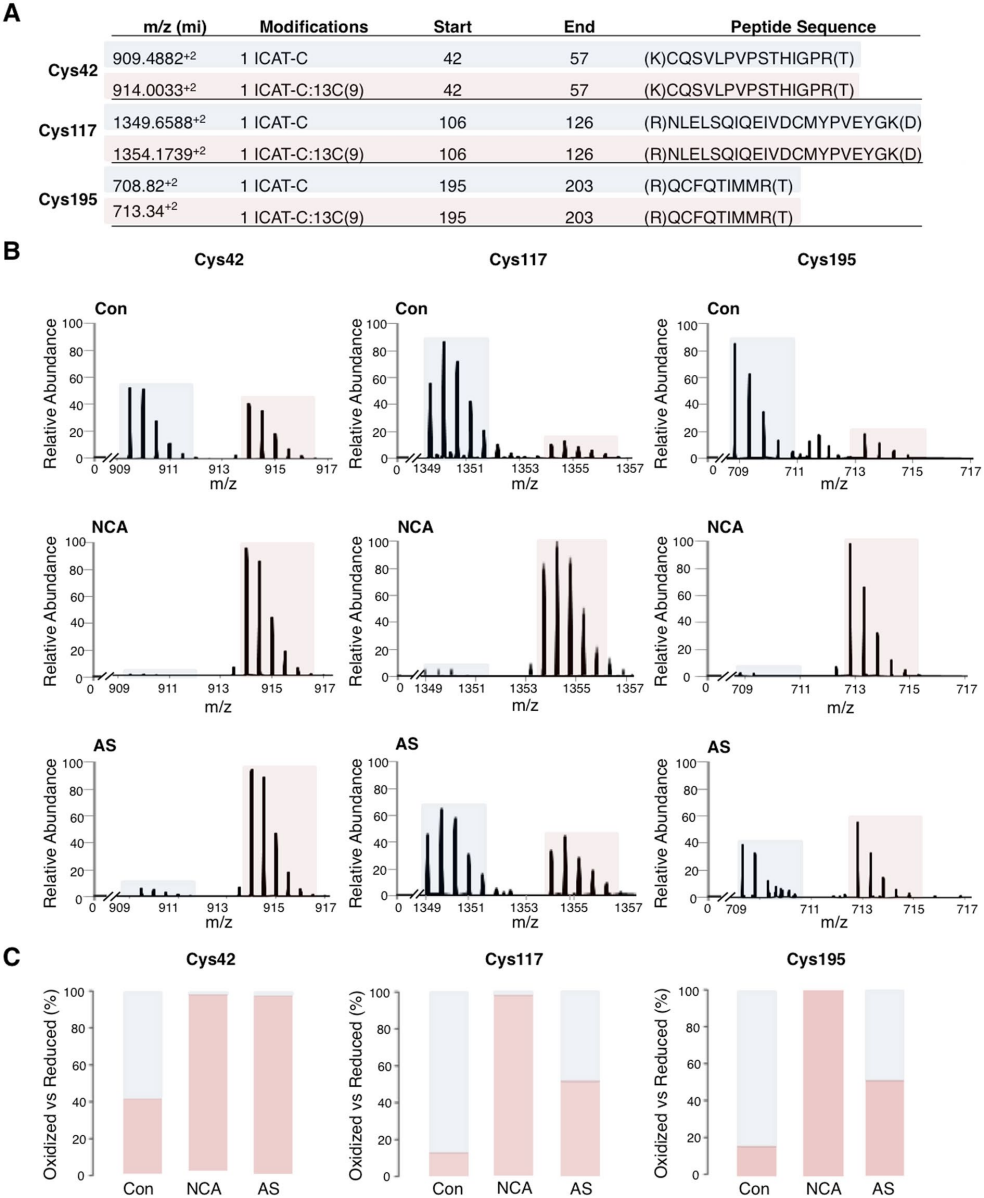
Identification of HNO-modified cysteines in PKGIα by NOxICAT. (**A**) Exposure of recombinant human PKGIα to HNO donors induced oxidation of Cys42, Cys117 and Cys195 in PKGIα. The table summarises the mass charge (m/z) in molecular ions (mi) of the detected peptides containing the cysteines in the reduced (blue) and oxidised (pink) state. (**B**) Fourier Transform Mass Spectrometry spectra are expressed as the relative abundance of the detected peptides in m/z containing Cys42 (left), Cys117 (middle) and Cys195 (right), vehicle-treated (top), after exposure to 1-nitrosocyclohexyl acetate (NCA; 25 mmol/L, 15 min) (middle) or Angeli’s salt (AS; 25 µmol/L, 15 min) (bottom). (**C**) Quantification of the detected oxidised (pink) versus reduced (blue) peptide fraction is shown as percent of the total detected peptides.

**Figure 2:**
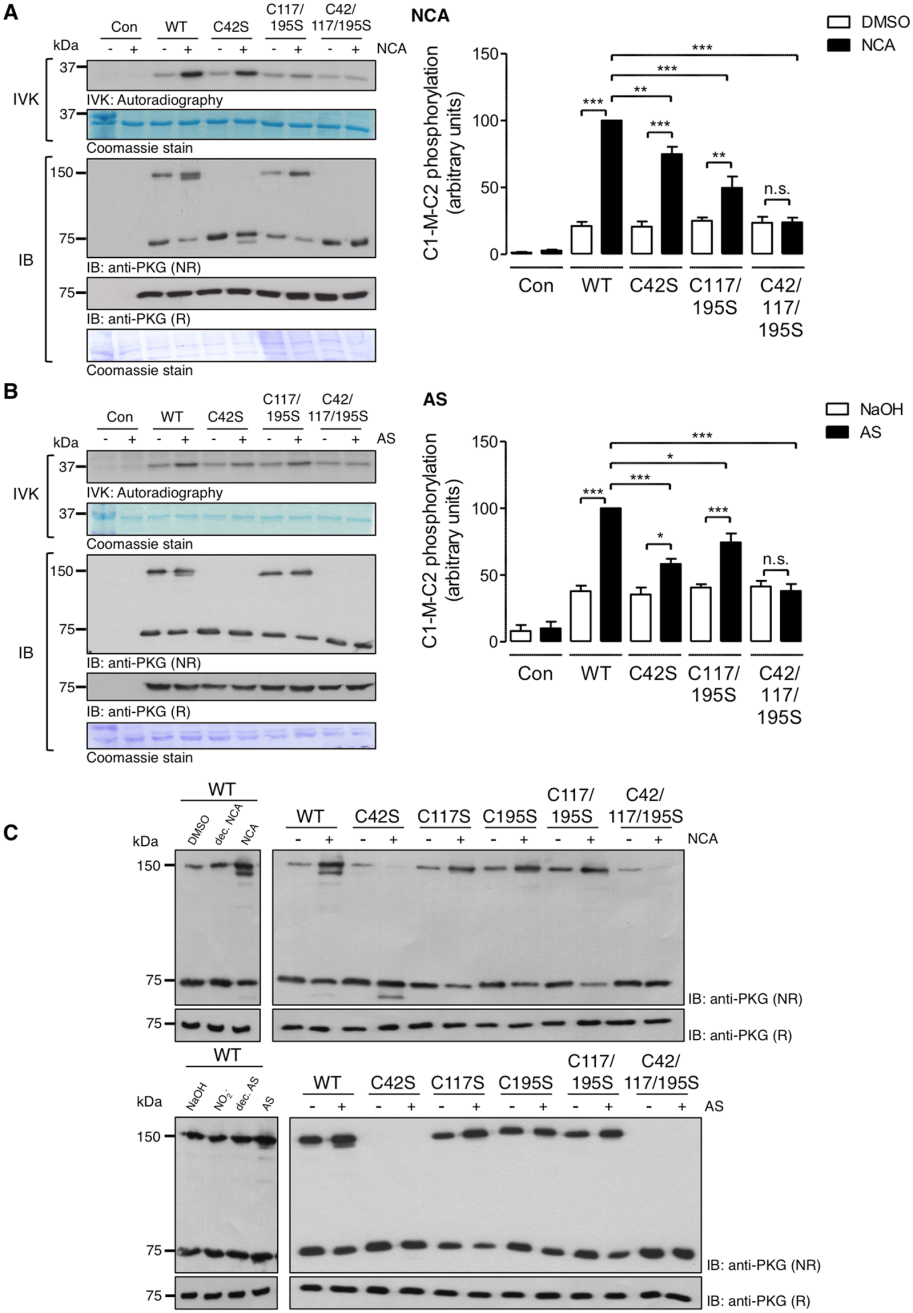
Effect of HNO-mediated PKGIα oxidation on kinase activity. HEK-293 cells were transfected to express PKGIα WT or various mutants Cys42Ser, Cys117/195Ser, Cys42/117/195Ser, exposed to (**A**) NCA (100 µmol/L, 30 min) or (**B**) AS (500 µmol/L, 15 min). *In vitro* kinase (IVK) assays were performed in cell lysates by addition of recombinant His_6_-tagged C1-M-C2 domain of cardiac myosin-binding protein C as a substrate in the presence of γ^32^P-ATP. Substrate phosphorylation was detected by autoradiography (figure shows cropped version, full representation in online supplement). Bar charts represent the results of 5 independent experiments. Data are expressed in comparison to the WT response after HNO treatment. *P < 0.05, **P < 0.01, ***P < 0.001 by comparison against each respective unstimulated control or WT after HNO-exposure. Western immunoblot analysis for PKGIα under non-reducing (NR) or reducing (R) conditions was performed in the same samples (figure shows cropped reducing blots, full representation in online supplement). Representative immunoblots show PKGIα migrating at 75 kDa (monomer) and 150 kDa (dimer). Data are representative of 5 independent experiments. (**C**) Analysis of HNO-induced oxidation of PKGIα. HEK-293 cells were transfected to express PKGIα WT or various mutants Cys42Ser, Cys117Ser, Cys195Ser, Cys117/195Ser, Cys42/117/195Ser, exposed to NCA (100 µmol/L, 30 min, upper panel) or the respective controls namely DMSO or decomposed NCA donor compound or AS (500 µmol/L, 15 min, bottom panel) or the respective controls NaOH, nitrite (NO_2_^−^) or decomposed AS. Western immunoblot analyses for PKGIα under non-reducing (NR) or reducing (R) conditions were performed. Representative immunoblots show PKGIα migrating at 75 kDa (monomer) and 150 kDa (dimer) under non-reducing and at 75 kDa under reducing conditions. Data are representative of 5 independent experiments. n.s.: non-significant; dec: decomposed.

To investigate whether PKGIα is a direct HNO target and is modified by cysteine oxidation, recombinant PKGIα was exposed to two chemically distinct HNO donors, namely 1-nitrosocyclohexyl acetate (NCA) or Angeli’s salt (AS). The oxidation state of cysteines in PKGIα was then analysed by NOxICAT methodology, involving differential isotopic labeling of reduced or oxidised thiol groups, and subsequent mass spectrometry. Without prior reduction with DTT, recombinant PKGIα is fully oxidised by air. A DTT concentration-response curve was performed initially with recombinant PKGIα (Supplementary Figure 1A,B) to determine the optimal DTT concentration. In the NOxICAT experiments, we observed increased oxidation of Cys42, Cys117 and Cys195 in PKGIα after exposure to either donor (Fig. 1). Despite DTT-pretreatment, Cys42 was still found partially oxidized in control samples (Fig. 1B, left panel). However, upon treatment with NCA or AS, Cys42 was detected exclusively in the oxidised form. Cys117 as well as Cys195 were predominantly found in the reduced state under basal conditions, but were completely oxidised after NCA treatment (Fig. 1B, middle and right panels); AS induced oxidation of these cysteines as well, albeit to a lesser extent than NCA (Fig. 1B, middle and right panels). The fact that Cys42 was almost completely oxidised by both donors suggests that HNO induces Cys42 interdisulfide formation between PKGIα monomers. The similar extent of Cys117 and Cys195 oxidation following AS or NCA treatment was consistent with the possibility of the formation of an intradisulfide bond between these two cysteines. Indeed, an intradisulfide bond between them within the cGMP-binding pocket of PKGIα has been reported, but was considered a constitutive modification^24^. To investigate the potential impact of intradisulfide formation after HNO-exposure on kinase activity, wildtype (WT), Cys42Ser, Cys117/195Ser or Cys42/117/195Ser PKGIα that cannot form the interdisulfide, intradisulfide or either, respectively, were expressed in HEK-293 cells. Kinase activity and oxidation status of PKGIα after exposure to HNO donors was analysed by assessing substrate phosphorylation with radiolabeled γ^32^P-ATP and autoradiography or western immunoblotting under non-reducing conditions (Fig. 2). The activity of WT PKGIα was significantly increased in response to both HNO donors as reflected by enhanced phosphorylation of recombinant C1-M-C2 domain of cardiac myosin-binding protein C, an established substrate of PKGIα^42^ (Fig. 2A,B top panels IVK, bar charts). Replacement of Cys42 or Cys117/195 by oxidation-resistant serine significantly reduced substrate phosphorylation in response to each of the HNO donors, whilst expression of the Cys42/117/195Ser mutant completely abolished substrate phosphorylation. This is consistent with the involvement of Cys42, Cys117 and Cys195 in HNO-mediated enhancement of PKGIα activity. The same samples were analysed by western immunoblotting under non-reducing conditions to investigate the PKGIα oxidation status. Cells expressing WT PKGIα displayed the monomer migrating at 75 kDa, as well as the interprotein disulfide dimer at 150 kDa. Interestingly, after exposure to NCA (Fig. 2A) or AS (Fig. 2B), which increased interdisulfide formation, two additional bands were observed. One band ran just below the monomer and the other just below the interdisulfide form. Such faster migrating bands on non-reducing gels are typical for proteins that form an intradisulfide^44, 45^, which rationally in this case occurs between Cys117 and Cys195. In cells expressing Cys42Ser PKGIα, no interdisulfide formation was detectable with only the monomer and the intradisulfide band below the monomer evident in response to HNO treatment. Importantly, replacement of either Cys117 or Cys195 was sufficient to abolish the appearance of the lower bands (Fig. 2C), again consistent with the formation of an intradisulfide between Cys117 and Cys195. After replacement of all three cysteines, only monomeric PKGIα was detectable after HNO treatment. Addition of 2-mercaptoethanol to the sample buffer reduced the interdisulfide-linked dimer and also the faster migrating bands (Fig. 2A,B, bottom panels). This provided yet further corroboration of HNO inducing an intradisulfide in PKGIα. None of the control compounds used in this study (DMSO, decomposed NCA, NaOH, NO_2_^−^, decomposed AS) induced significant oxidation of PKGIα detectable by western immunoblot analysis (Fig. 2C, left panels).

**Figure 3:**
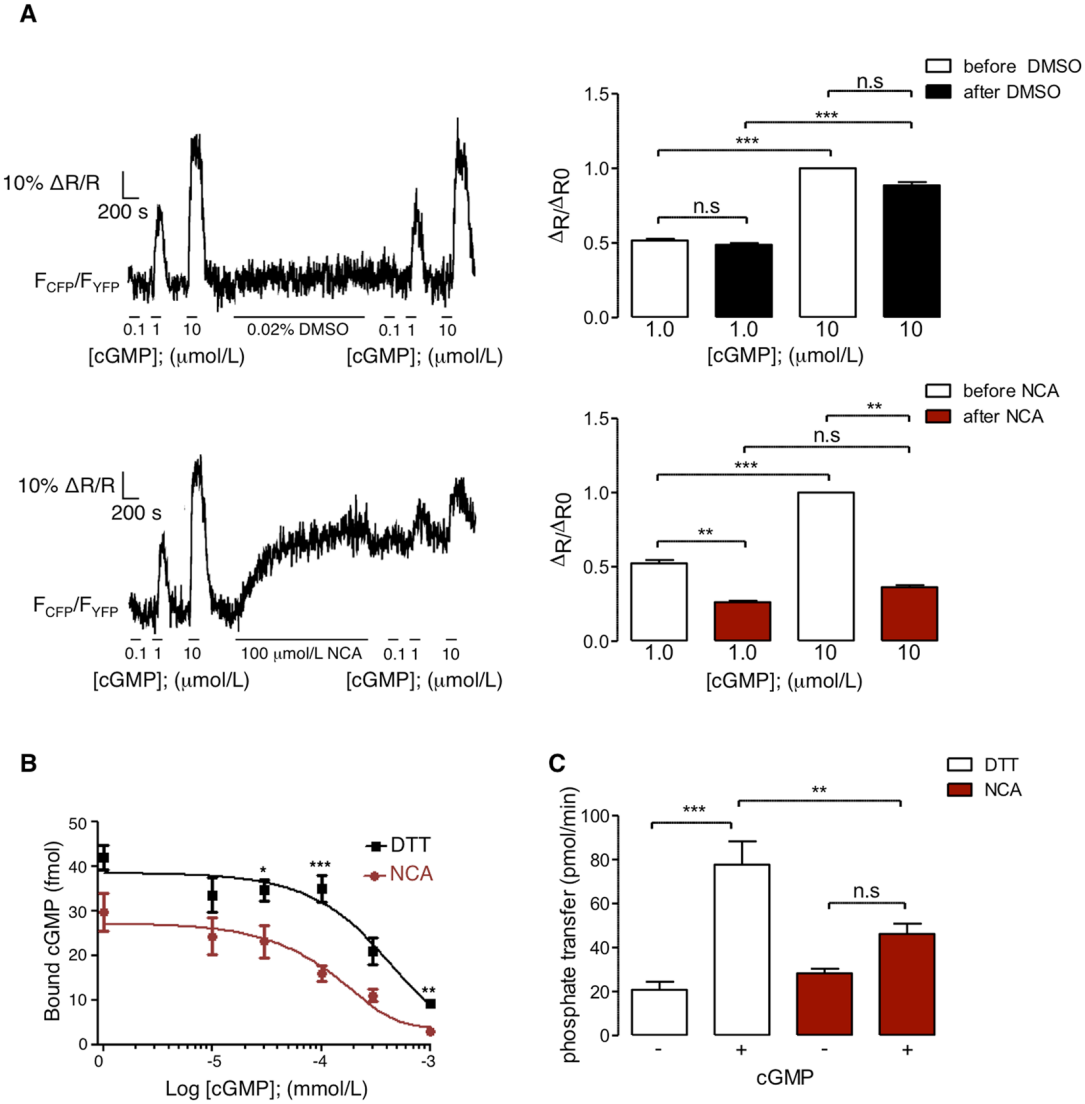
Assessment of intradisulfide-induced changes in the cGMP-binding domain of PKGIα. (**A**) β-Escin-permeabilised primary mouse VSMCs stably expressing the FRET sensor cGi500 were superfused with intracellular-like medium (ICM) containing increasing concentrations of cGMP (0.1, 1, 10 µmol/L). This was followed by incubation with DMSO (0.02%; upper panel) or NCA (100 µmol/L; bottom panel) and by another incubation with ICM supplemented with increasing concentrations of cGMP (0.1, 1, 10 µmol/L). Changes of the FRET signals were recorded by epifluorescence microscopy. Representative FRET traces are shown on the left. The bar charts on the right summarise the FRET results from 10 cells per group as the ΔR/ΔR0 (amplitude relative to the signal induced by the first application of 10 µmol/L cGMP) induced by cGMP incubation before (white bars) or after exposure to DMSO (black; upper panel) or NCA (red; bottom panel). **P < 0.01, ***P < 0.001, comparing cGMP-induced changes in FRET ratio before and after DMSO or NCA by two-way ANOVA. (**B**) *In vitro* cGMP binding to PKGIα after exposure to DTT (100 mmol/L, 10 min) (black squares) or NCA (100 µmol/L, 30 min) (red dots) was investigated by measuring binding of (^3^H)cGMP in the presence of increasing concentrations of unlabeled cGMP (0, 10, 30, 100, 300, 1000 nmol/L). The data are representative of 5 independent experiments. *P < 0.05, **P < 0.01, ***P < 0.001 comparing exposure to DTT or NCA at the same cGMP concentrations. (**C**) To assess PKGIα activity, *in vitro* kinase assays were performed using γ^32^P-ATP after DTT (100 mmol/L, 10 min) or NCA-treatment (100 µmol/L, 30 min), in the presence or absence of cGMP (300 nmol/L) with Glasstide as a substrate. Bar chart summarises data of 6 independent experiments. PKGIα activity was expressed as phosphotransfer into PKGIα substrate per minute. **P < 0.01, ***P < 0.001 intergroup comparison; n.s.: non-significant.

Next, the influence of NCA-induced oxidation of Cys117 and Cys195 on the conformation of PKGIα was assessed. This analysis was performed in primary mouse vascular smooth muscle cells (VSMCs), stably expressing the Förster resonance energy transfer (FRET)-based cGMP biosensor cGi500 (cGMP indicator with an EC_50_ of 500 nmol/L cGMP). The cGi500 contains the two cGMP-binding sites of PKGIα including Cys117 and Cys195 and reports conformational changes upon cGMP binding via changes in FRET efficiency^37, 38^. We reasoned that cGi500 FRET might also be altered by NCA-induced oxidation of Cys117 and Cys195 in its cGMP-binding domain. To limit possible side effects of NCA application to intact VSMCs, resulting in activation of sGC and cGMP synthesis from intracellular GTP, VSMCs were permeabilised with β-escin to keep the intracellular concentrations of GTP and cGMP low. Moreover, experiments with the sGC blocker ODQ were performed to further exclude the possibility that NCA-induced FRET changes were caused by activation of sGC and subsequent cGMP generation (data not shown). As a positive control, permeabilised VSMCs were exposed to increasing concentrations of cGMP. As expected, the FRET signals showed concentration-dependent changes upon exposure to cGMP, with no effect of the vehicle solvent DMSO (0.02%; Fig. 3A, top panels). In the absence of cGMP, exposure of the permeabilised cells to NCA alone was sufficient to induce a strong change of the sensor’s FRET response (Fig. 3A, lower left panel). These results suggested that NCA-induced intradisulfide formation in the cGMP-binding domain of PKGIα is associated with a conformational change that mimics cGMP binding. In line with this model, subsequent superfusion of NCA-treated cells with increasing cGMP concentrations produced much smaller FRET changes than the same cGMP concentrations before NCA application (Fig. 3A, lower panels). Based on this, it is likely that intracellular PKGIα can also be activated in a cGMP-independent manner by HNO-induced intradisulfide formation.

To further substantiate these findings, cGMP-binding assays were performed *in vitro*. Purified bovine PKGIα was reduced by dithiothreitol (DTT; Fig. 3B; black line) or oxidised by NCA (Fig. 3B; red line). (^3^H)-cGMP binding to PKGIα was assessed in the presence of increasing amounts of unlabeled cGMP (Fig. 3B). This revealed significantly reduced cGMP binding to the NCA-oxidised kinase when compared to that reduced by DTT. To investigate whether the NCA-induced modifications of PKGIα modulate cGMP-induced changes in kinase activity, *in vitro* kinase assays were performed in the presence or absence of cGMP. Purified bovine PKGIα was reduced with DTT (Fig. 3C; open bars) or oxidised with NCA (Fig. 3C; red bars). In the absence of cGMP, PKGIα displayed basal kinase activity. Addition of cGMP to DTT-PKGIα significantly enhanced kinase activity 3.7 fold versus no cGMP. This cGMP-induced potentiation of PKGIα-activity was not observed in the NCA-oxidised kinase. These results are in accordance with the data obtained by FRET analysis and suggest that intradisulfide formation by NCA reduces subsequent cGMP binding to the kinase concluding that NCA-induced modification of PKGIα activates the kinase, but delimits its further activation by subsequent cGMP addition.

**Figure 4:**
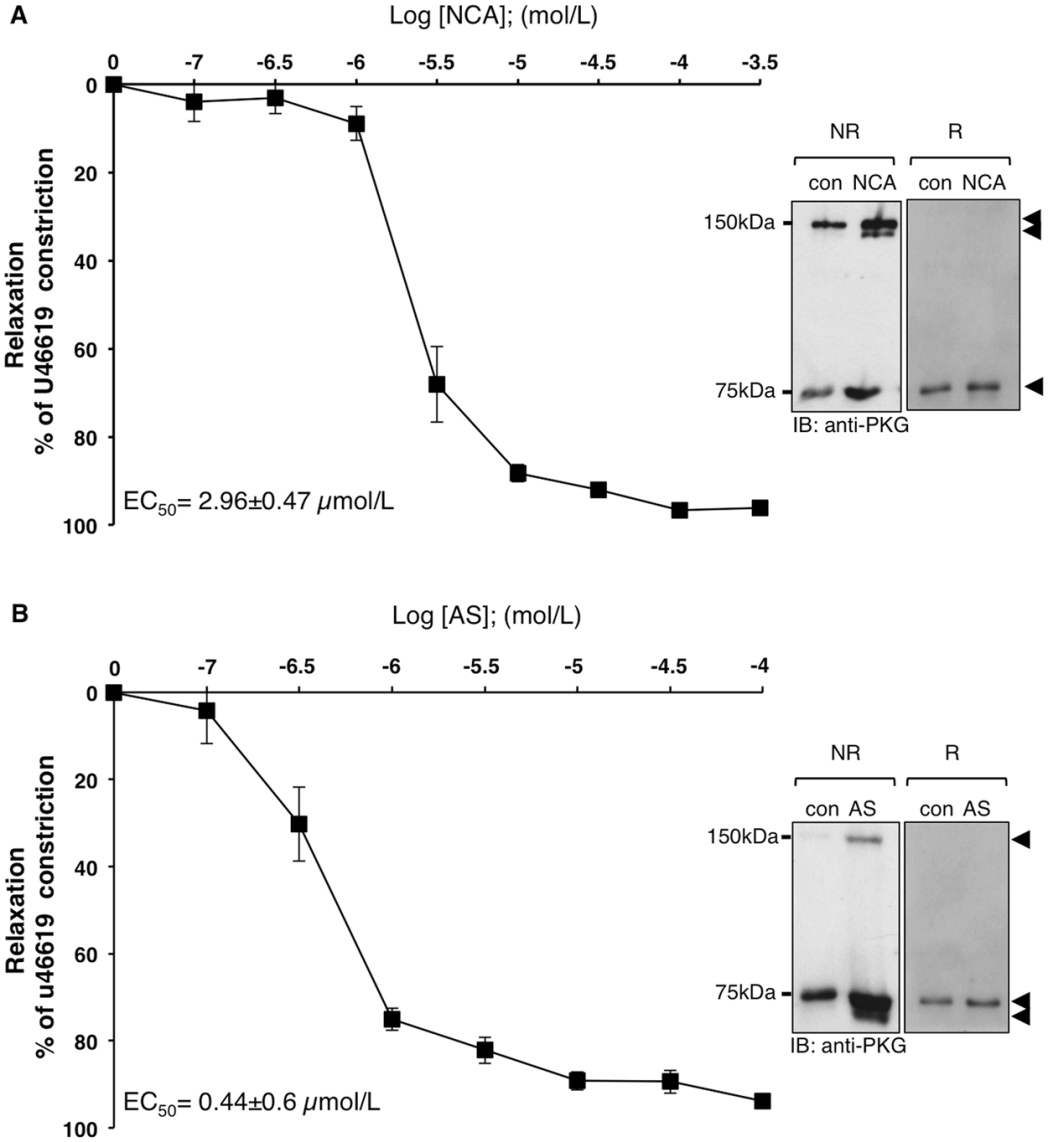
Correlation of intradisulfide formation in endogenous PKGIα with HNO-mediated vasorelaxation *in vitro*. The effect of NCA (**A**) or AS (**B**) on vasorelaxation was assessed in mesenteric arteries from wildtype (WT; black) mice. U46619 (100 nmol/L) was used to preconstrict vessels and then increasing concentrations of NCA (0.1, 0.3, 1, 3, 10, 30, 100, 300 µmol/L) or AS (0.1, 0.3, 1, 3, 10, 30, 100 µmol/L) were administered. Experiments were performed in 1-2 vessels derived from at least 4 animals for each HNO donor. Western immunoblot analysis for PKGIα was performed under reducing (R) or non-reducing (NR) conditions in vessel homogenates exposed to 30 µmol/L NCA or 3 µmol/L AS.

**Figure 5:**
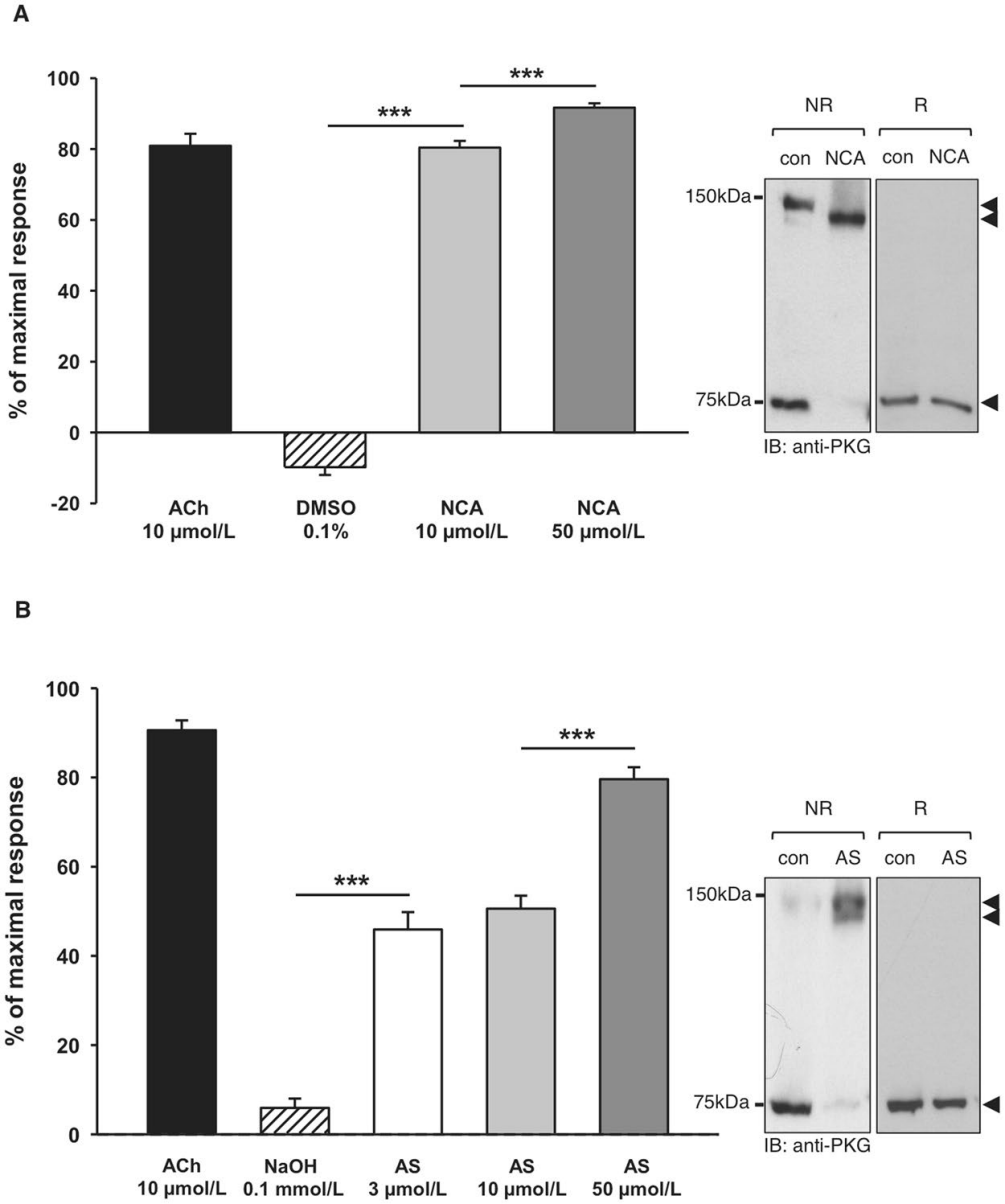
Correlation of intradisulfide formation in endogenous PKGIα with HNO-mediated vasorelaxation *in vivo*. Both HNO donors induced concentration-dependent dilations in arterioles *in vivo*. (**A**) The effect of NCA was studied in 68 vessels from 6 mice. (**B**) The effect of AS was studied in 73 vessels from 6 mice. Data are given as mean ± SEM. *** indicates *P* < 0.001 for paired comparisons (*t*-test). Western immunoblot analysis for PKGIα was performed under reducing (R) and non-reducing (NR) conditions in isolated cremaster muscles exposed to 50 µmol/L NCA or AS. Black arrows indicate the positions of monomeric (75 kDa), inter- (150 kDa) and intradisulfide (below 150 kDa) PKGIα.

The correlation between HNO-mediated intradisulfide formation in endogenous PKGIα and vasorelaxation was investigated in isolated murine mesenteric arteries *in vitro* and in the skeletal muscle microcirculation *in vivo* in WT mice. Both HNO donors induced concentration-dependent vasorelaxation in mesenteric arteries (NCA: EC_50_ = 2.96 ± 0.47 µmol/L; AS: EC_50_ = 0.44 ± 0.6 µmol/L; 1–2 vessels from 4 mice), which was paralleled by enhanced inter- and intradisulfide formation in PKGIα as evidenced by western immunoblot analysis under non-reducing conditions (Fig. 4A,B, right panel). Similar concentration-dependent vasorelaxation was observed after exposure of the microcirculation to HNO donors *in vivo* (Fig. 5A NCA; n = 68 arterioles in 6 mice; Fig. 5B AS; n = 73 arterioles in 6 mice). Arterioles studied here exhibited maximal diameters between 13 and 49 µm and their means were not different between the intervention groups (NCA: 27.6 ± 0.8 µm; AS: 29.2 ± 0.9 µm). Vessels exhibited spontaneous tone (calculated as the quotient of resting and maximal diameter) amounting to 40 ± 1% (ranging from 10 to 85%). This spontaneous constriction level was similar in both groups (NCA: 41 ± 2%; AS: 40 ± 2 of maximal diameter; *P* = 0.63). The endothelium-dependent dilator acetylcholine (10 µmol/L) induced in both groups a significant dilation (NCA: 82 ± 3%; AS: 91 ± 2 of maximal response) assuring intact endothelial function and dilator capacity. Exposure to 10 µmol/L NCA dilated the arterioles from 11.0 ± 0.9 to 24.6 ± 0.8 µm. This dilation was further enhanced after exposure to 50 µmol/L NCA (26.3 ± 0.8 µm; *P* < 0.05 vs. 10 µmol/L NCA). The solvent alone (0.1% DMSO) resulted in a small constriction (from 11.4 ± 0.8 to 10.0 ± 0.6 µm; *P* < 0.05) (Fig. 5A). Exposure to 3 µmol/L AS dilated the arterioles from 11.6 ± 0.8 to 19 ± 0.8 µm and the highest concentration used (50 µmol/L) dilated the vessels to 23.8 ± 0.8 µm. The solvent alone (0.1 mmol/L NaOH) induced a small dilatation (from 11.6 ± 0.8 to 19.6 ± 0.8 µm) (Fig. 5B). *In vivo* vasorelaxation by both HNO donors was accompanied by oxidation of PKGIα into both its inter- and intradisulfide form in this tissue as shown in the western immunoblots under non-reducing conditions (Fig. 5A,B, right panels).

## Discussion

**Figure 6:**
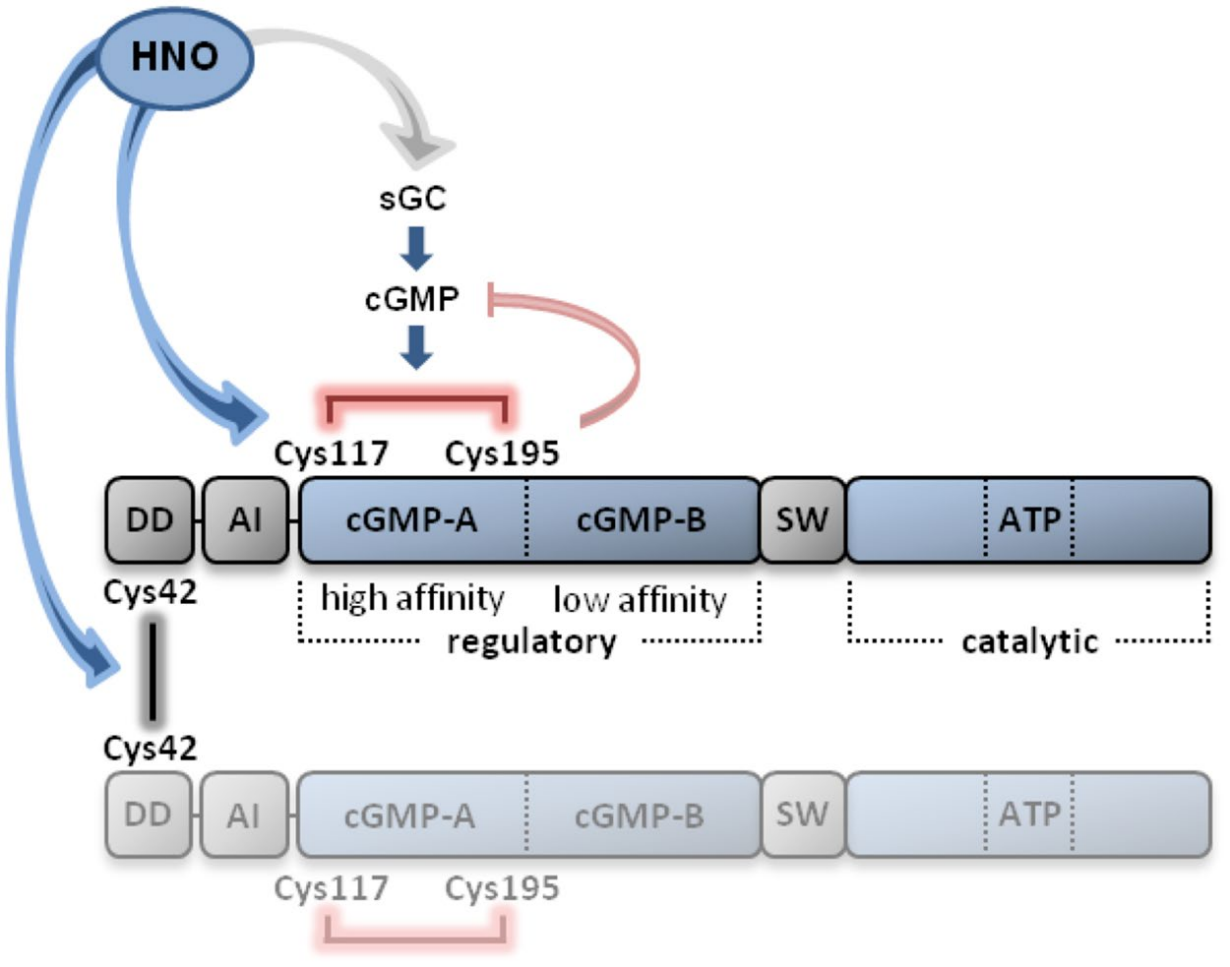
Illustration of the domain structure of the PKGIα homodimer and the localisation of cysteines that are modified by exposure to HNO donors to form inter- and intradisulfide bonds. DD: docking and dimerisation domain; AI: autoinhibitory domain; cGMP-A: high affinity cGMP-binding domain; cGMP-B: low affinity cGMP-binding domain; SW: switch helix; ATP: ATP-binding domain; sGC: soluble guanylate cyclase.

Oxidation of Cys42 in PKGIα has been reported as a mechanism that regulates protein kinase activity potentially by modulating substrate targeting, independently of the second messenger cGMP^23^. This discovery has highlighted a role for oxidants in the regulation of important physiological functions such as blood pressure^45, 46^. The main finding of the present study is the description of a novel molecular mechanism for the regulation of catalytic PKGIα activity through oxidative modifications by HNO. HNO oxidised Cys42, together with Cys117 and Cys195 in PKGIα, resulting in inter- and intradisulfide formation, respectively (Fig. 6). The intradisulfide, which localises within the high affinity cGMP-binding site directly activates PKGIα and as such mimics second messenger-achieved activation. In this connection, the intradisulfide is not cooperative with cGMP-dependent activation, as the oxidation lowers the cGMP affinity of the kinase, thus uncoupling it from the classical mode of activation.

PKGIα contains 11 cysteines^47^, some of which have been described previously as redox sensors^23, 24^. Whilst interprotein disulfide formation via Cys42 has been extensively investigated and characterised^23, 25, 30, 45, 46^, information regarding the impact of Cys117 and Cys195 oxidation on PKGIα functions is scarce. Intraprotein disulfide bond formation was shown previously to occur after exposure of PKGIα to metal-ion induced oxidative stress *in vitro* ^24^. This intradisulfide, which forms in the high affinity cGMP-binding site and therefore may be considered to be in a more rational position for regulating kinase activity than the Cys42 in the leucine zipper domain, was also observed in a crystal structure of the regulatory domain of PKGIα^31^. This would be consistent with a constitutive disulfide. Indeed, in these studies from the Dostmann laboratory, it was suggested that this intradisulfide most likely mediated the oxidative activation of the kinase. However, it is clear from the observations reported here that cellular PKGIα is predominantly reduced under basal conditions, with the disulfide only accumulating when oxidant levels are elevated. It is likely that the intradisulfide can be observed crystallographically in recombinant protein, because the kinase is removed from cellular thiol-reducing buffers, such as thioredoxin or glutathione, that maintain solvent accessible cysteinyl thiols in their reduced state. Indeed, interprotein disulfides in PKGIα or protein kinase A RI were originally reported as constitutive structural bonds^48, 49^, but subsequently were shown to be absent basally without pro-oxidant interventions^23, 50^. Recently, S-guanylation of Cys195 by nitro-cGMP was reported as a novel modification of PKGIα, leading to persistent kinase activity^51^. As S-guanylation occurs at reduced thiols, this provides independent corroboration of the observations made here that the intradisulfide is largely not present in the absence of pro-oxidant conditions.

By using a differential isotope-based thiol-trapping mass spectrometry-based approach, we identified oxidative modification of Cys42, Cys117 and Cys195 in PKGIα in response to HNO. This approach has the advantage of providing quantitative information about the redox state of individual cysteines. These data showed Cys42 was susceptible to oxidation also under control conditions, whilst Cys117 and Cys195 only oxidised upon addition of HNO. Although the matching levels of oxidation of Cys117 and Cys195 caused by NCA or AS treatment is consistent with structural studies showing that PKGIα forms an intradisulfide at this location^31^, NOxICAT analysis cannot provide a formal demonstration of this modification. However, inter- or intradisulfides can be assessed by migration changes using non-reducing western immunoblotting, as the oxidations induce higher or lower molecular weight gel shifts, respectively that can be normalised by reducing agents^44, 52^. Indeed, non-reducing western immunoblot analysis of cell homogenates supported the NOxICAT data, with a basal level of Cys42 interdisulfide detected under control conditions, whereas the intradisulfide only formed upon oxidant-exposure. It is notable that the intradisulfide induced by HNO had low stoichiometry, but this is because this intervention will concomitantly elevate cGMP, which as shown here competes with the formation of the intradisulfide. It is evident that the interplay of mechanisms that control PKGIα activity is complex especially as cGMP binding or Cys42 oxidation also reciprocally negatively regulates each other. Another layer of complexity is added by the observation that cGMP binding negatively influences oxidation of Cys42^28, 46, 53^. A significant finding of this study is that the interdisulfide formed at Cys42 attenuated the formation of the intradisulfide, as Cys42Ser PKGIα showed markedly potentiated intradisulfide formation in response to HNO. This complex array of interrelated, interacting and modulating mechanisms may serve as a feedback that delimits over-activation or over-recruitment of the kinase to regulatory stimuli.

In addition to the molecular detection and characterisation, it was important to investigate potential functional effects of nascent intradisulfide on PKGIα activity. This was achieved using *in vitro* kinase assays performed on lysates from HEK-293 cells that were transfected with WT PKGIα or various ‘redox-dead’ mutants of PKGIα in the presence or absence of HNO donors. An obvious conclusion from this experiment was that both HNO donors activated the WT kinase, but the activation was impaired in the various cysteine mutants that are resistant to inter- or intradisulfide formation. The overall important conclusion to reiterate is that the primary novel finding of this work is, namely that PKGIα forms an intradisulfide and this activates the kinase. This conclusion is rationally based on the structure and is consistent with S-guanylation also activating the kinase^31, 51^. Perhaps either of these oxidative activation mechanisms that target Cys195 disrupts the interaction of the autoinhibitory domain with the catalytic domain to enable catalytic competence. This is in essence similar to what cGMP achieves when it binds to its high affinity-binding site that also contains this redox active cysteine. Interestingly, the HNO donors used in our study showed apparent differences concerning their impact on kinase activity. NCA-induced kinase activity was almost completely abolished in the intradisulfide-deficient mutant, whilst activity after AS treatment was greatly reduced in the Cys42Ser mutant. These results are in accordance with the NOxICAT results showing that NCA and AS modify the same spectrum of cysteines in PKGIα, albeit to a different extent. Both donors oxidise the highly susceptible Cys42, however, NCA was superior to AS with regard to oxidation of Cys117 and Cys195. One explanation for the discrepant oxidation efficiency of the HNO donors may relate to their different kinetics of HNO release. NCA has a half-life of 2 hours^32^, whereas it is only 2 to 5 minutes for AS^54^. Releasing initially large amounts of HNO by AS may facilitate self-consumption, potentially by reducing the amount of HNO available for target oxidation. Alternative explanations could relate to the release of byproducts released by the two different HNO donors. When AS decomposes into HNO an equivalent of nitrite and a small amount of NO^55^ is also generated, whereas NCA releases HNO and a thiol-reactive moiety of its scaffold^33^, each of them exerts biological effects that have to be taken into account^56^. Distinct behavior with regard to target oxidation and functional effects induced between different HNO donors has in fact been described previously^32^. However, as both donors induce the same post-translational modification and induce the same biological action, it is logical to conclude that this is likely due to HNO release, especially as this is the only common characteristic of these compounds.

FRET experiments performed in VSMCs isolated from transgenic mice constitutively expressing a FRET-biosensor comprising the cGMP-binding sites of PKGIα, allowed the impact of HNO-induced intradisulfide on PKGIα activity to be defined in a cellular context and compared to that induced by cGMP. Indeed, exposure to HNO increased the FRET response in the absence of cGMP, suggesting activation of the kinase. This activation was comparable to that achieved by cGMP-dependent stimulation and substantiates that the kinase is activated by intradisulfide formation and is consistent with the modification relieving the autoinhibition of the catalytic domain. Exposure to increasing concentrations of cGMP did not potentiate kinase activity induced by HNO, consistent with the intradisulfide limiting cGMP binding. Future experiments involving recombinant mutant PKGIα would be valuable in establishing whether the difference in cGMP-binding to PKGIα is definitely mediated by oxidant-induced intradisulfide formation.

The HNO-mediated intradisulfide formation in PKGIα that is reported here most likely contributes mechanistically to the previously described impact of HNO on blood pressure reduction. This is supported by the fact that HNO induces significant amounts of intradisulfide in endogenous PKGIα. This activates the kinase, and so rationally significantly underlies the correlation between vasorelaxation and oxidation of endogenous PKGIα observed in two different vascular beds *in vivo*.

Taken together, our study describes a novel oxidative mechanism for the activation of PKGIα by HNO. Our observations are consistent with the intradisulfide inducing the direct activation of the kinase, essentially mimicking cGMP binding, with the interdisulfide mediating substrate targeting as it was recently shown^29^. This explains why disrupting either of these disulfides by mutagenesis impairs oxidant-induced kinase activity and substrate phosphorylation.

## Electronic supplementary material

Supplementary Figures
