# Supplementary material for: Oxidant sensor in the cGMP-binding pocket of PKGIα regulates nitroxyl-mediated kinase activity
Source: Sci Rep. 2017 Aug 30;7:9938. doi: 10.1038/s41598-017-09275-1 (PMC5577323; doi:10.1038/s41598-017-09275-1)

Oxidant sensor in the cGMP-binding pocket of PKGIα regulates nitroxyl-mediated kinase activity

Sonia Donzelli[a,b,♯,*], Mara Goetz[a,b,*], Kjestine Schmidt[c], Markus Wolters[d], Konstantina Stathopoulou[a,b], Simon Diering[a,b], Oleksandra Prysyazhna[e], Volkan Polat[a,b], Jenna Scotcher[e], Christian Dees[f], Hariharan Subramanian[g,b], Elke Butt[f], Alisa Kamynina[e], Sophie Schobesberger[a,b], S. Bruce King[h], Viacheslav O. Nikolaev[g,b], Cor de Wit[c], Lars I. Leichert[i], Robert Feil[d], Philip Eaton[e,1], Friederike Cuello[a,b,1]

[a]Department of Experimental Pharmacology and Toxicology, Cardiovascular Research Center, University Medical Center Hamburg-Eppendorf, Martinistrasse 52, 20246 Hamburg, Germany.
[b]DZHK (German Center for Cardiovascular Research), partner site Hamburg/Kiel/Lübeck, University Medical Center Hamburg-Eppendorf, Martinistrasse 52, 20246 Hamburg, Germany.
[c]Institute of Physiology, University of Lübeck, Ratzeburger Allee 160, 23562 Lübeck, Germany.
[d]Interfaculty Institute of Biochemistry, University of Tübingen, Hoppe-Seyler-Str. 4, 72076 Tübingen, Germany.
[e]King´s College London, Cardiovascular Division, British Heart Foundation Centre of Excellence, the Rayne Institute, St Thomas´ Hospital, London SE17EH, United Kingdom.
[f]Institute of Experimental Biomedicine II, University Medical Center Würzburg, Grombühlstraße 12, 97080 Würzburg, Germany.
[g]Institute of Experimental Cardiovascular Research, University Medical Center Hamburg-Eppendorf, Martinistrasse 52, 20246 Hamburg, Germany.
[h]Department of Chemistry, Wake Forest University, Winston-Salem, North Carolina 27109, USA.
[i]Institute of Biochemistry and Pathobiochemistry - Microbial Biochemistry, Ruhr University Bochum, Universitätsstrasse 150, 44780 Bochum, Germany.
[♯]present address: Laboratory of Molecular and Chemical Biology of Neurodegeneration, Brain Mind Institute, Ecole Polytechnique Fédérale de Lausanne (EPFL), 1015 Lausanne, Switzerland
[*]joint first author [1]joint corresponding author

Supplementary Figure 1:

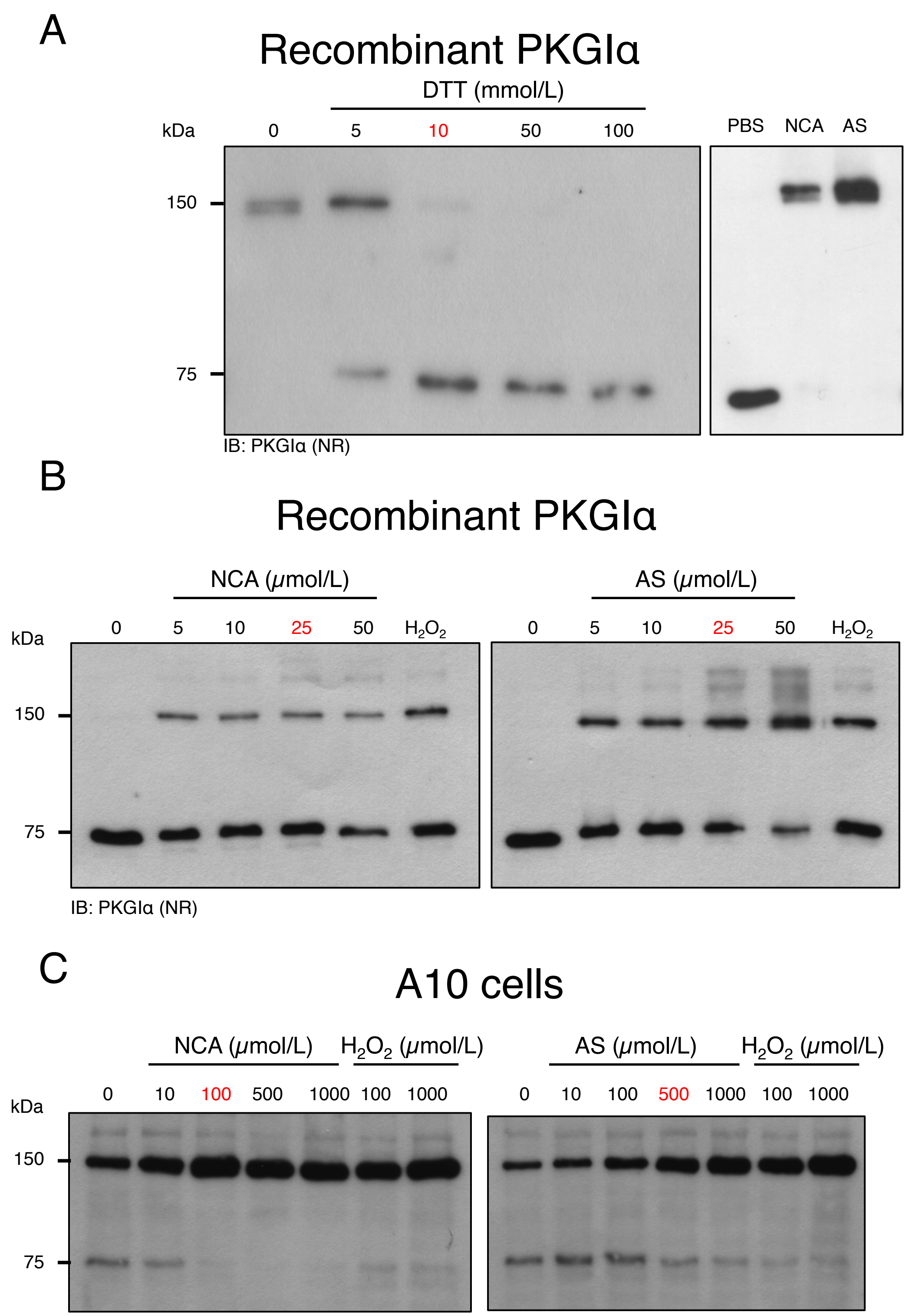

Suppl. Fig. 1. (**A**) Recombinant PKGIα was exposed to increasing concentrations of DTT (0-100 mmol/L) for 10 min (left panel), buffer exchange performed and subsequently exposed to vehicle (PBS), NCA (25 µmol/L) or AS (25 µmol/L) for 15 min and western immunoblot analysis performed under non-reducing conditions. (**B**) Recombinant PKGIα was pretreated with 10 mmol/L DTT for 10 min and subsequently exposed to increasing concentrations of NCA (0-50 µmol/L), AS (0-50 µmol/L) or H_2O_2 (100 µmol/L) for 15 min. (**C**) Rat vascular smooth muscle cells (A10) were exposed to increasing concentrations of NCA (0-1000 µmol/L; 30 min), AS (0-1000 µmol/L; 15 min) or 100-1000 µmol/L H_2O_2 for 10 min, cells harvested and western immunoblot analysis performed under non-reducing gel conditions for PKGIα.

Supplementary information: full blots for Figure 2

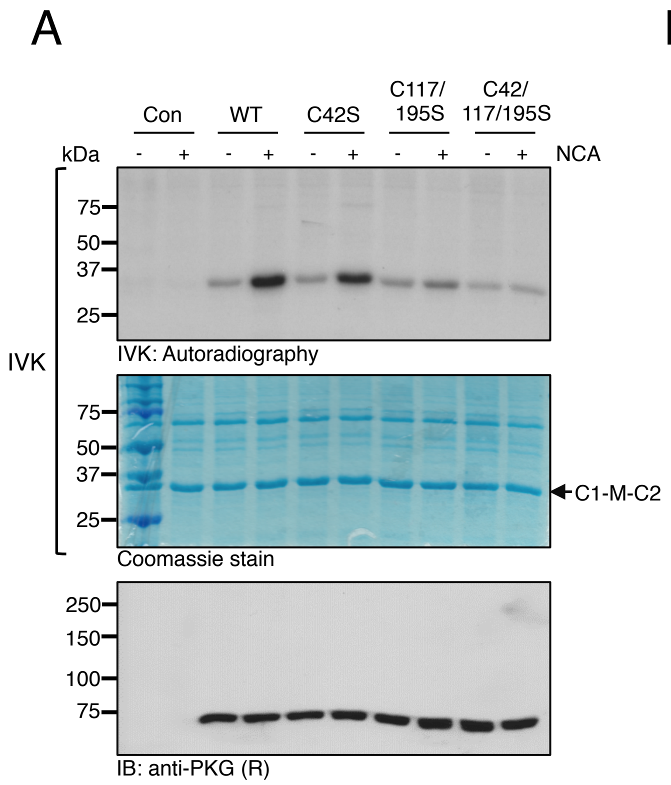

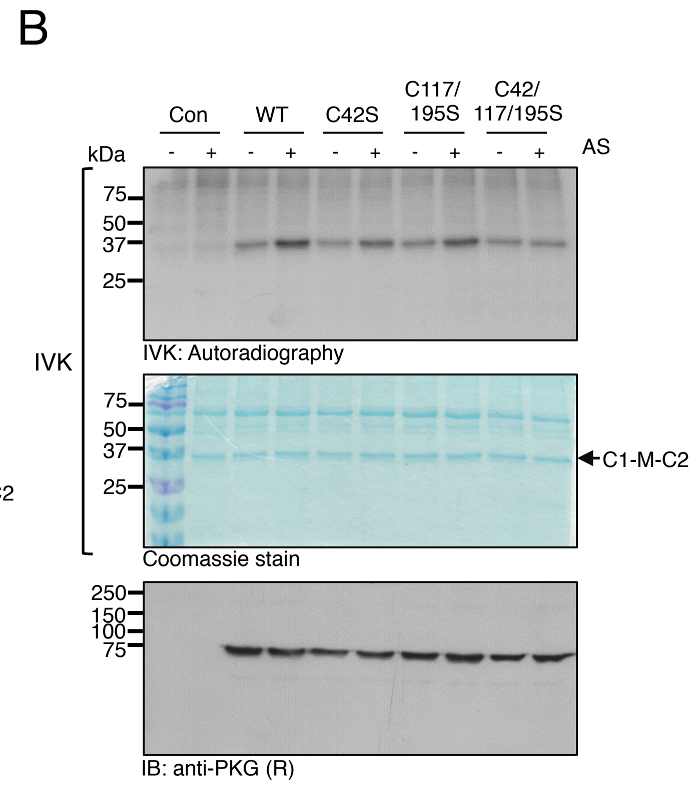

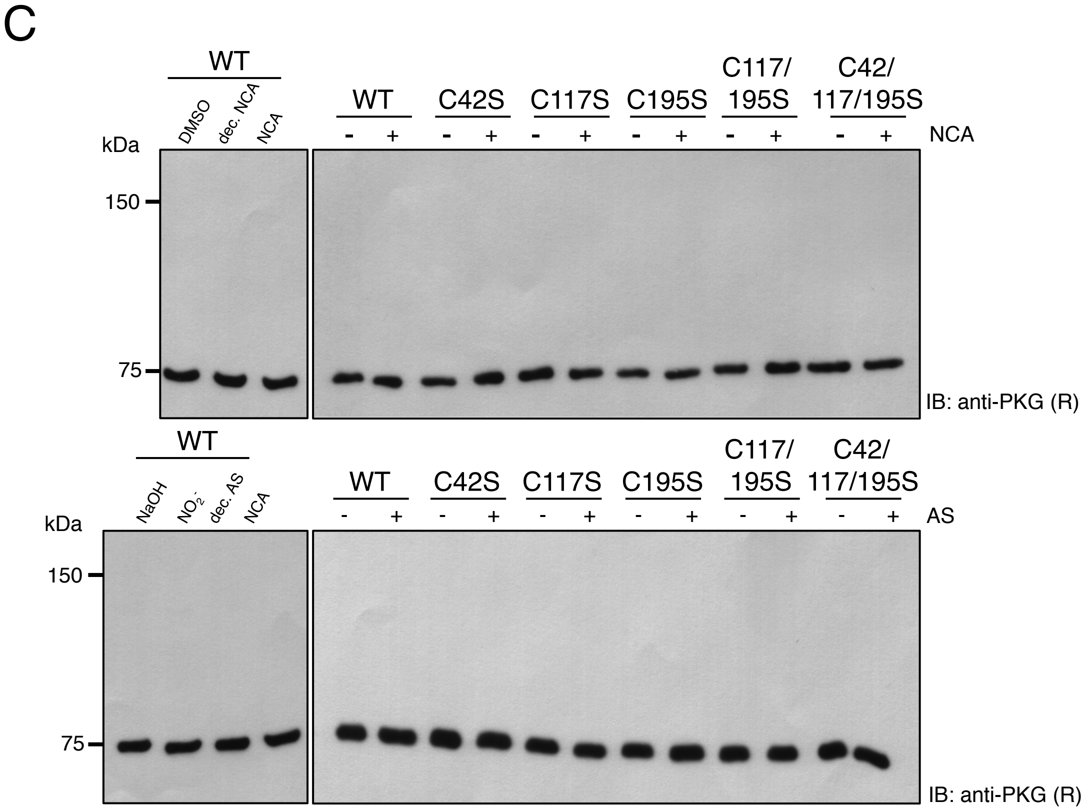

Supplement: Supplementary file 1 — Supplementary Figures [file 41598_2017_9275_MOESM1_ESM.pdf]
